# Progressive Hemorrhage and Myotoxicity Induced by *Echis carinatus* Venom in Murine Model: Neutralization by Inhibitor Cocktail of N,N,N',N'-Tetrakis (2-Pyridylmethyl) Ethane-1,2-Diamine and Silymarin

**DOI:** 10.1371/journal.pone.0135843

**Published:** 2015-08-14

**Authors:** Ankanahalli N. Nanjaraj Urs, Chandrasekaran Ramakrishnan, Vikram Joshi, Kanve Nagaraj Suvilesh, Teregowda Veerabasappa Gowda, Devadasan Velmurugan, Bannikuppe Sannanaik Vishwanath

**Affiliations:** 1 Department of Studies in Biochemistry, University of Mysore, Manasagangotri, Mysuru, Karnataka, India; 2 Centre of Advanced Study in Crystallography and Biophysics, University of Madras, Guindy Campus, Chennai, Tamil Nadu, India; 3 Bioinformatics Infrastructure Facility, University of Madras, Guindy Campus, Chennai, Tamil Nadu, India; Universidade Federal do Rio de Janeiro, BRAZIL

## Abstract

Viperbite is often associated with severe local toxicity, including progressive hemorrhage and myotoxicity, persistent even after the administration of anti-snake venom (ASV). In the recent past, investigations have revealed the orchestrated actions of Zn^2+^ metalloproteases (Zn^2+^MPs), phospholipase A_2_s (PLA_2_s) and hyaluronidases (HYs) in the onset and progression of local toxicity from the bitten site. As a consequence, venom researchers and medical practitioners are in deliberate quest of potent molecules alongside ASV to tackle the brutal local manifestations induced by aforesaid venom toxins. Based on these facts, we have demonstrated the protective efficacy of inhibitor cocktail containing equal ratios of N,N,N’,N’-tetrakis (2-pyridylmethyl) ethane-1,2-diamine (TPEN) and silymarin (SLN) against progressive local toxicity induced by *Echis carinatus* venom (ECV). In our previous study we have shown the inhibitory potentials of TPEN towards Zn^2+^MPs of ECV (IC_50_: 6.7 μM). In this study we have evaluated *in vitro* inhibitory potentials of SLN towards PLA_2_s (IC_50:_ 12.5 μM) and HYs (IC_50:_ 8 μM) of ECV in addition to docking studies. Further, we have demonstrated the protection of ECV induced local toxicity with 10 mM inhibitor cocktail following 15, 30 min (for hemorrhage and myotoxicity); 60 min (for hemorrhage alone) of ECV injection in murine model. The histological examination of skin and thigh muscle sections taken out from the site of ECV injection substantiated the overall protection offered by inhibitor cocktail. In conclusion, the protective efficacy of inhibitor cocktail is of high interest and can be administered locally alongside ASV to treat severe local toxicity.

## Introduction

Over the past decade, systemic approach of drug discovery has identified combinatorial therapy as a new approach [1]. As ideal synergistic combination of potent molecules provides increased curative efficacy and decreased drug toxicity, this approach is gaining popularity over highly specific single component therapies [2]. The combinatorial drug approach followed the successful precedent to treat tuberculosis, microbial infections and antiretroviral treatment for HIV [3–4]. In this post-genomic era combinatorial drug approach is a happening agenda in sepsis and cancer [5–6]. Local toxicity induced by venomous viperid or crotalid bites is never the less a pathological condition, caused by mixture of toxins rather than a single toxin present in the venom. Hence, treatment of venom induced progressive tissue damage, that persists even after anti-snake venom (ASV) administration is still a challenging issue for the existing strategies of snakebite management [7].

At the outset Zn^2+^ metalloproteases, also called as snake venom metalloproteases (SVMPs) were mainly blamed for such complications, as they degrade proteins of extra cellular matrix (ECM), basement membrane and blood coagulation cascade, resulting in a wide range of hemostatic alterations and local tissue damage [8]. However, in the recent past, besides SVMPs, other key hydrolytic enzymes such as snake venom phospholipase A_2_s (SVPLA_2_s) and snake venom hyaluronidases (SVHYs) are also known to induce local tissue damage [7, 9–11]. SVPLA_2_s can be catalytically active forms (Asp49) or inactive variants (Lys49). Enzymatically active forms of PLA_2_s cause hydrolysis of membrane phospholipids, whereas enzymatically inactive variants of PLA_2_s act via perturbation of the membrane and hence, in both the cases, alteration of membrane structure occurs which further leads to the onset of edema, hemorrhage and myotoxicity [12]. On the other hand, SVHYs though recognized as minor enzymes, aid diffusion of venom toxins from the site of bite to systemic circulation by hydrolyzing the dermal barrier, long chain glycosaminoglycan-hyaluronic acid and thus potentiating the local toxicity of venoms which are rich in hemorrhagic toxins [13]. In this regard, progressive local toxicity of viper bites can be attributed to the orchestrated actions of SVMPs, SVPLA_2_s and SVHYs popularly known as hemorrhagic complex [14].

In our previous study, we have shown the neutralization abilities of N,N,N’,N’-tetrakis (2-pyridylmethyl) ethane-1,2-diamine (TPEN)—a specific Zn^2+^ chelating agent against the proteolytic, hemorrhagic and myotoxic activities of *Echis carinatus* venom metalloproteases (ECVMPs). The affinity of TPEN towards free Zn^2+^ is reported in femto molar concentration (0.67 fM). However, higher concentrations of TPEN were used to achieve inhibition of Zn^2+^ metalloproteases (6.7 μM) and for preventing the tissue degrading potentials (20 mM) of crude *E*. *carinatus* venom (ECV) [15]. This clearly suggests that targeting single venom toxin inducing local tissue damage will be effective only towards purified toxins. However, to target progressive tissue damage induced by orchestrated action of mixture of toxins in crude venom, ideal combination of inhibitors which are specific and can work in a combinatorial fashion are essential. The concomitant inhibition of these enzymes not only decreases the magnitude of local tissue damage but also prevents the diffusion of systemic toxins into circulation, thereby increasing the survival time of snakebite victims. Keeping this in mind, in addition to TPEN, in this study we have screened and evaluated silymarin (SLN) as an effective *E*. *carinatus* venom phospholipase A_2_s (ECVPLA_2_s) and *E*. *carinatus* venom hyaluronidases (ECVHYs) inhibitor. Further, the inhibitor cocktail of TPEN and SLN has been systematically evaluated for its effectiveness towards protection of progressive hemorrhage and myotoxicity upon independent injections following ECV administration and the results obtained are presented below.

## Materials and Methods

### Venom

Lyophilized powder of ECV was procured and used in experiments as described in our previous publication [15]

### Chemicals

^14^C-oleic acid was obtained from Perkin Elmer Life Sciences Inc. (Boston, USA). Scintillation cocktail (Ultima Gold) was obtained from Packard Bioscience Co. (Meriden, USA). Alcian blue, 6-O-palmitoyl-L-ascorbic acid (AP), *Escherichia coli* [lyophilized cells of strain W (ATCC9637)], fatty acids, *n*-acetyl glucosamine (NAG), *p*-dimethylaminobenzaldehyde (*p*-DMAB), pentobarbital sodium salt, silymarin (SLN), and N,N,N',N'-tetrakis (2-pyridylmethyl) ethane-1,2-diamine (TPEN) were purchased from Sigma Aldrich (Saint Louis, USA). Hyaluronic acid was purchased from Across Organics (New Jersey, USA). BSA, dimethyl sulfoxide (DMSO), ethanol (HPLC grade), and water (HPLC grade), were purchased from Sisco Research Laboratories (Mumbai, India). All other chemicals and reagents used in this study were of analytical grade and were procured from local firms (Mysore, India).

### Experimental animals

Adult Swiss Albino mice weighing 25–30 g were used for pharmacological studies. Animals were collected from University Central Animal Facility and housed under a controlled environment. All experimental protocols were approved by the Institutional Animal Ethical Committee (No: UOM/IAEC/25/2011), University of Mysore, Mysuru, and were in accordance with the guidelines of the Committee for the Purpose of Control and Supervision of Experiments on Animals (CPCSEA). Control, venom injected and treatment groups were monitored regularly (5 min intervals) until the end of incubation period and there were no unintended death of animals. After incubation, animals were euthanized by administering pentobarbital sodium salt (30 mg/kg; i.p.) and dissection was performed.

### Computational studies

To assess the inhibitory potentials of aristolochic acid (AA), 6-O-palmitoyl-L-ascorbic acid (AP), oleanolic acid (OA), and ursolic acid (UA) against ECVPLA_2_s; cromolyn sodium salt (CSS), sodium aurothiomalate hydrate (SAH), and silymarin (SLN) against ECVHYs induced fit docking (IFD) [16] analysis was employed. The crystal structure of a calcium bound monomeric form of ECVPLA_2_ (PDB ID: 1OZ6) [17] was retrieved from the protein data bank (PDB) [18–19]. Whereas, the structure of the ECVHY was modeled as the experimentally determined structure was unavailable. ECVHY was modeled using the sequence of *E*. *ocellatus* venom hyaluronidase (UniProt ID: A3QVN2) [20] retrieved from the UniProt protein sequence database [21]. The template structures of bee venom hyaluronidase (PDB ID: 1FCQ) [22] and human hyaluronidase (PDB ID: 2PE4) [23] were used for modeling as they showed 33.3% and 42% sequence identity and 92% and 70% query coverage, respectively with the target sequence. The 3D structure of ECVHY was built using modeller software [24–25] with the Chimera interface. The discrete optimized protein energy and Ramachandran plot [26] were used to choose and validate the final model. Crystal structure of ECVPLA_2_ and predicted homology model of ECVHY were prepared using "protein preparation wizard" of the Schrodinger software as it facilitates assignment of proper bond order, addition of charges, and protonation state prior to minimization. In addition, hydrogen bond network optimization and identification of proper ionization states (His tautomers) were also performed. Further, terminal angle of Asn, Gln, and His residues were allowed for 180° rotations and impref module was invoked for final constrained energy minimization with root mean square deviation cut off of 0.30 Å. The amino acid residues of ECVPLA_2_ (His48 and Asp49) and ECVHY (Thr19, Gln20, Leu44, Asp107, Glu109, Tyr180, Try227, Ala268, Trp302, and Ser304) were used to locate the active site for docking compounds of interest.

Ligands were downloaded from the PubChem structure database in 3D SDF format and prepared using the ligprep module [27]; pH 7±2 was set for ionization. Maximum of 32 stereo isomers and tautomers were allowed to generate for each ligand and chiralities of stereo isomers and low energy ring conformation were retained. For each ligand docking, maximum of 20 poses were obtained and the best of them was chosen based on the glide score, energy and the interactions with the active site residues. The Schrodinger software with OPLS_2005 force field [28] was used for all the calculations involved in ligand and protein preparations and docking.

### Phospholipase A_2_ activity

Phospholipase A_2_ (PLA_2_) activity was measured using ^14^C-oleate labeled autoclaved *E*. *coli* as substrate as described in [29]. For inhibition studies, similar reactions were carried out after pre-incubating 50 μg ECV with various concentrations of AP and SLN (0.1 nM-10 mM) for 15 min at 37°C. Inhibition was expressed as a percentage.

### Hyaluronidase activity

Hyaluronidase activity was carried out according to the method in [30] using hyaluronic acid as substrate, and activity was expressed in terms of *n*-acetyl glucosamine (NAG) released. For inhibition studies, hyaluronidase activity was determined after pre-incubating 100 μg ECV with various concentrations of AP and SLN (0.1 nM-10 mM) for 15 min at 37°C.

### Formulation of inhibitor cocktail of TPEN and SLN

TPEN and SLN stock solutions (200 mM each) were separately prepared in ethanol and DMSO respectively. Required concentration of inhibitor cocktail (0.3–30 mM; working standard) was formulated by mixing 1:1 molar concentrations of TPEN and SLN and final volume was adjusted using 50% ethanol. Aliquots were kept at 4°C until further use. After several repetitions and standardization experiments 1, 3, and 10 mM concentrations were used for inhibition studies.

### *In vivo* inhibition studies

Inhibition studies were carried out by ‘independent injection’ method (treatment mode) where, AP and SLN were separately used for edema inhibition. Whereas, different doses of inhibitor cocktail containing TPEN and SLN were used for the inhibition of hemorrhage, and myotoxicity at different time points following the administration of ECV.

### Edema-inducing activity

Edema-inducing activity of ECV was determined as described in [31]. For inhibition studies, similar experiments were carried out with different doses of AP and SLN following 15 min of 3 μg ECV administrations. The increase in weight due to edema was expressed as the ratio of the weight of edematous limb to the weight of normal (sham injected) limb X 100.

### Hemorrhagic activity

Experimental animals were randomly divided into different groups (n = 3) and hemorrhagic activity was determined as described in [32]. Inhibition studies were carried out by independently injecting different doses of inhibitor cocktail (in a total volume of 30 μl with saline) at different time points following 3 μg ECV administrations. Saline, ECV (3 μg), and inhibitor cocktail (0.3, 3 and10 mM) served as negative, positive and inhibitor controls respectively. Area of hemorrhage and histological changes at hemorrhagic spot were measured as described in our previous publication [15].

### Myotoxicity

Myotoxicity studies were performed according to the method in [33]. A group of mice (n = 3) were injected (i. m.) with 5 μg ECV in a total volume of 40 μl with saline. Mice which received saline alone served as control. For inhibition studies different doses of inhibitor cocktail were injected at different time points with appropriate controls following ECV injection. After 3 h of observation period, blood was collected by cardiac puncture to measure serum markers and thigh muscle tissue was processed for histological studies as described in our previous publication [15].

### Data analysis

The results of experiments were expressed as mean ± SD of three independent experiments performed in triplicate. Statistical analyses were carried out using Student’s *t*-test. The comparison between the groups were considered significant if *p* ≤ 0.05. *In vitro* and animal experimental data were analyzed using the statistical package Graph Pad Prism version 5.03 (La Jolla, USA) and docking analysis was performed using schrodinger software package (New York, USA).

## Results

### Computational studies for screening of potent inhibitors against ECVPLA_2_s and ECVHYs

**Fig 1:**
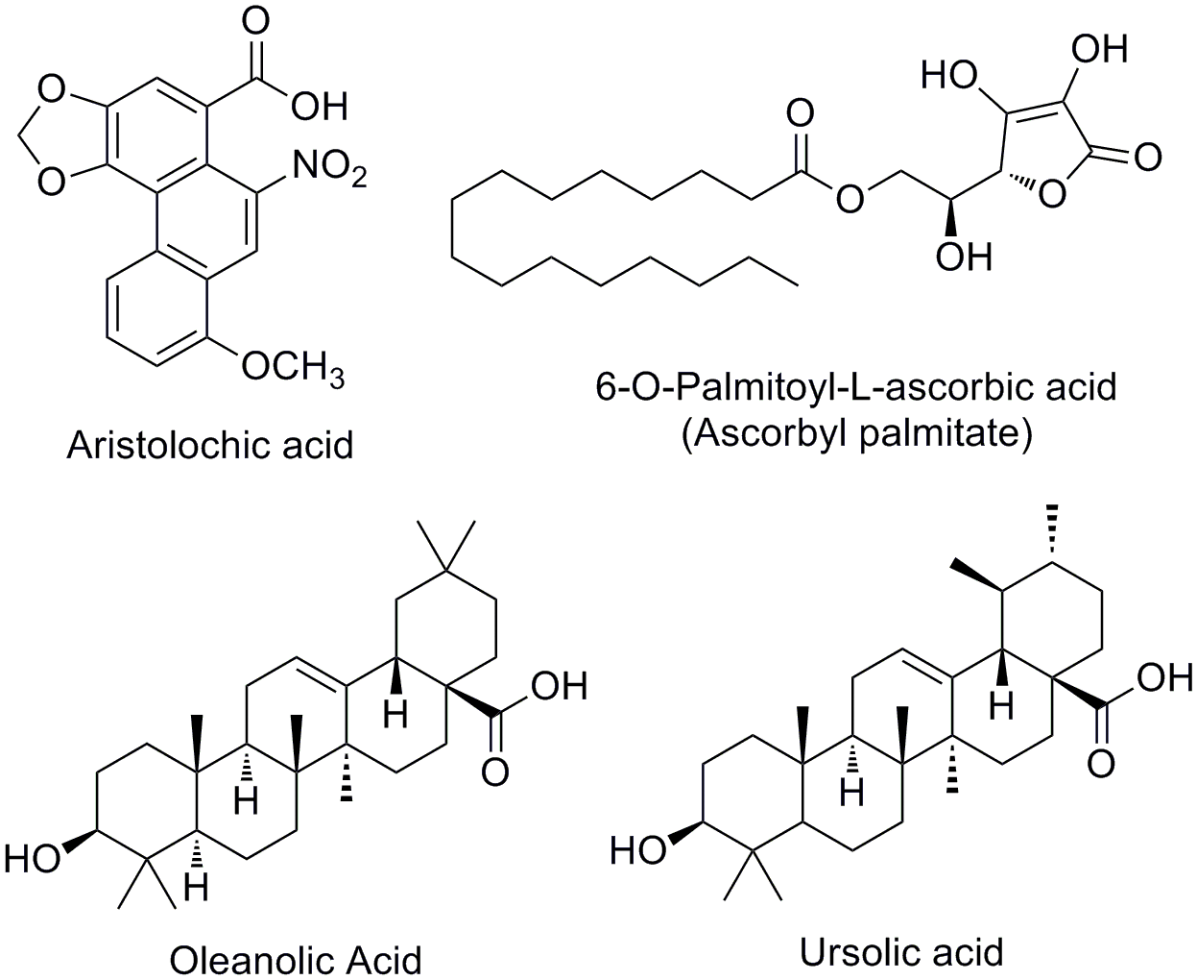
Phospholipases A_2_ inhibitors. Aristolochic acid (8-methoay-6-nitrophenanthro (3, 4-*d*) 1, 3-dioxole-S-carboxylic acid); ascorbyl palmitate; oleanolic acid; and ursolic acid (3*β*-hydroxy-urs-12-en-28-oic acid).

**Fig 2:**
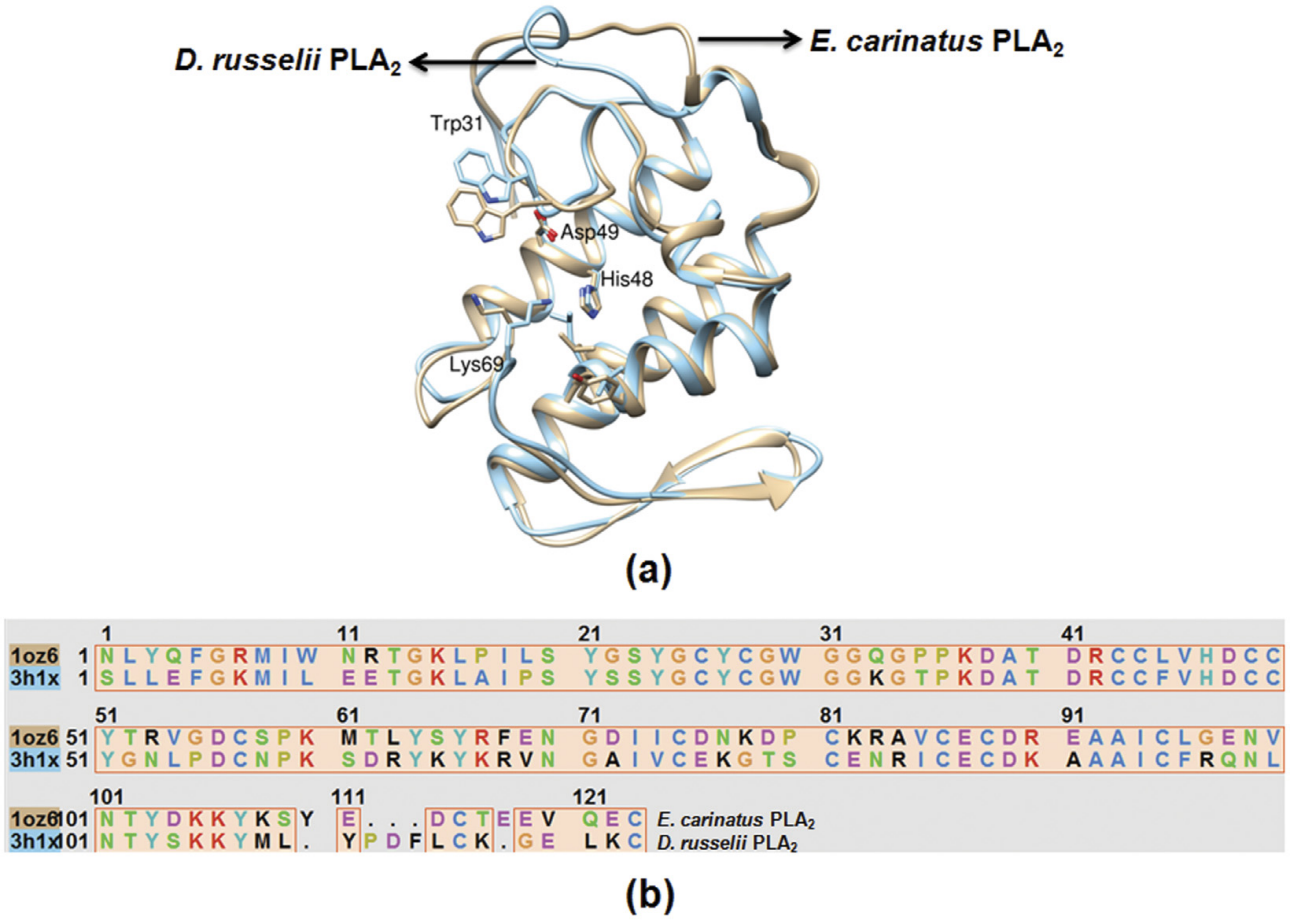
Multiple sequence alignment of *D*. *russelii* and *E*. *carinatus* venom sPLA_2_s. (a) Structure superposition shown as ribbon structure; (b) sequence alignment of sPLA_2_s. The sequences shares identical domain architecture with 57% identity, 70% similarity with His48, Asp49, Trp31, and Lys69 as conserved active site residues

**Fig 3:**
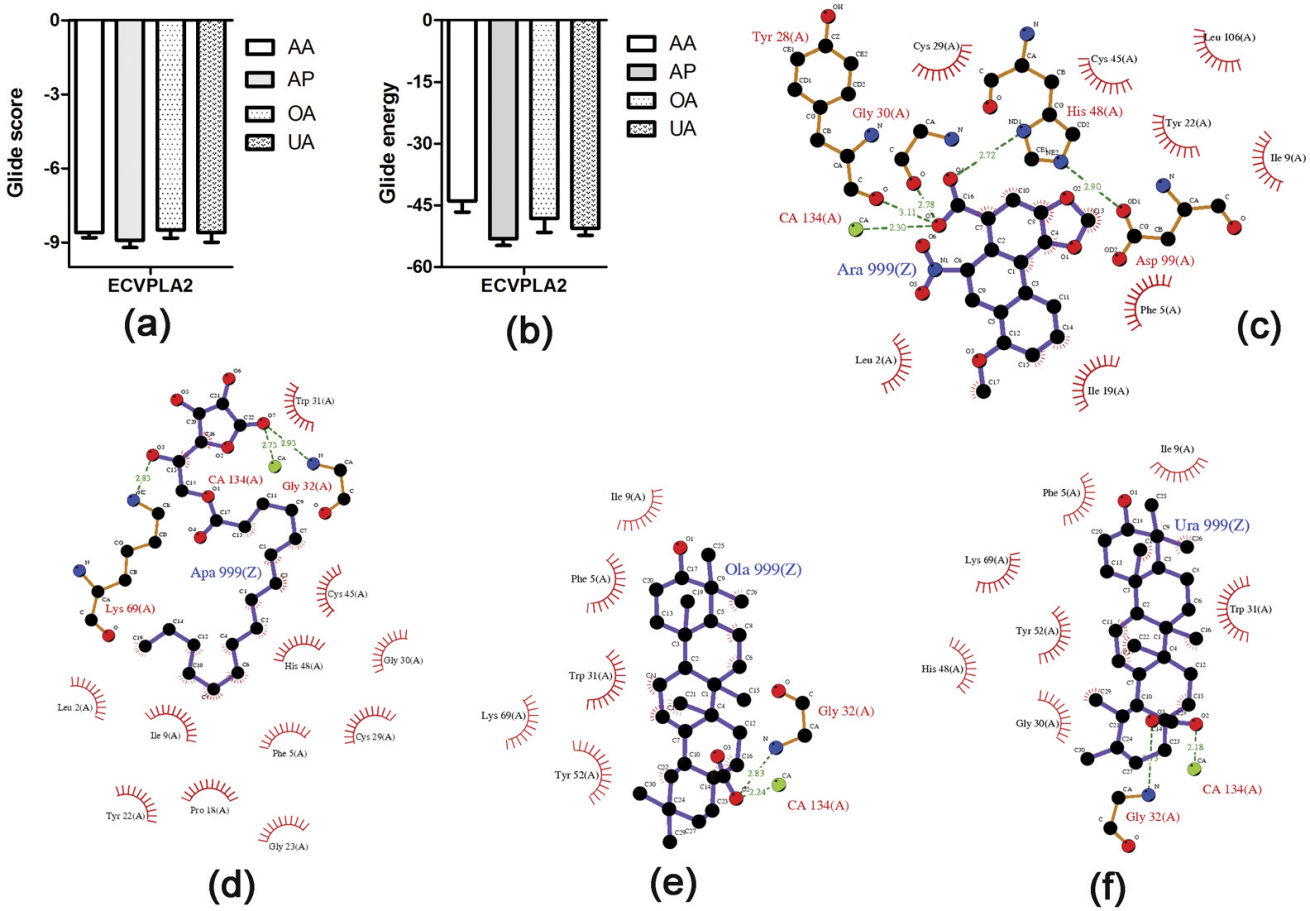
Energetically favorable binding modes of AA, AP, OA, and UA calculated using IFD method. Glide score (a) and glide energy (b) (calculated in kcal/mol) associated with best binding modes of AA, AP, OA, and UA with the active site of ECVPLA_2_. The hydrogen bonding and hydrophobic interactions between the enzyme and AA (c), AP (d), OA (e), and UA (f) respectively are depicted using the LigPlot software. AA, AP, OA, and UA are labeled using three letter codes “Ara”, “Apa”, “Ola”, and “Ura” respectively with a common residue number 999(Z).

Crystal structures of ECVPLA_2_, and 3D structures of inhibitor of interest were prepared as described in materials and methods and inhibitors were initially screened using IFD method. AA, AP, OA, and UA (Fig 1), previously reported from our team as potent *D*. *russelii* PLA_2_ inhibitors [34–37] were evaluated against ECVPLA_2_ as both of them are group IIa basic sPLA_2_s containing conserved calcium binding loop and catalytic network [38]. In addition, multiple sequence alignment of both the sequences revealed identical domain architecture with 57% identity, 70% similarity with His48, Asp49, Trp31 and Lys69 as conserved active site residues (Fig 2). Among the screened inhibitors AP (PubChem ID: CID 54680660) strongly interacted with ECVPLA_2_ giving steady output of interactions with active site residues. However, AA (PubChem ID: CID 2236), OA (PubChem ID: CID 10494), and UA (PubChem ID: CID 64945) exhibited different interactions on repeated trials. The steady hydrogen bond (Ca^2+^, Gly32, Lys69) and hydrophobic interactions (Trp31, Cys45, His48, Ile9, Phe5 and Leu2) of AP with ECVPLA_2_ was evidenced by its low glide score (-8.9 ± 0.3 kcal/mol) and glide energy (-53 ± 1.5 kcal/mol) compared to AA, OA and UA (Fig 3).

**Fig 4:**
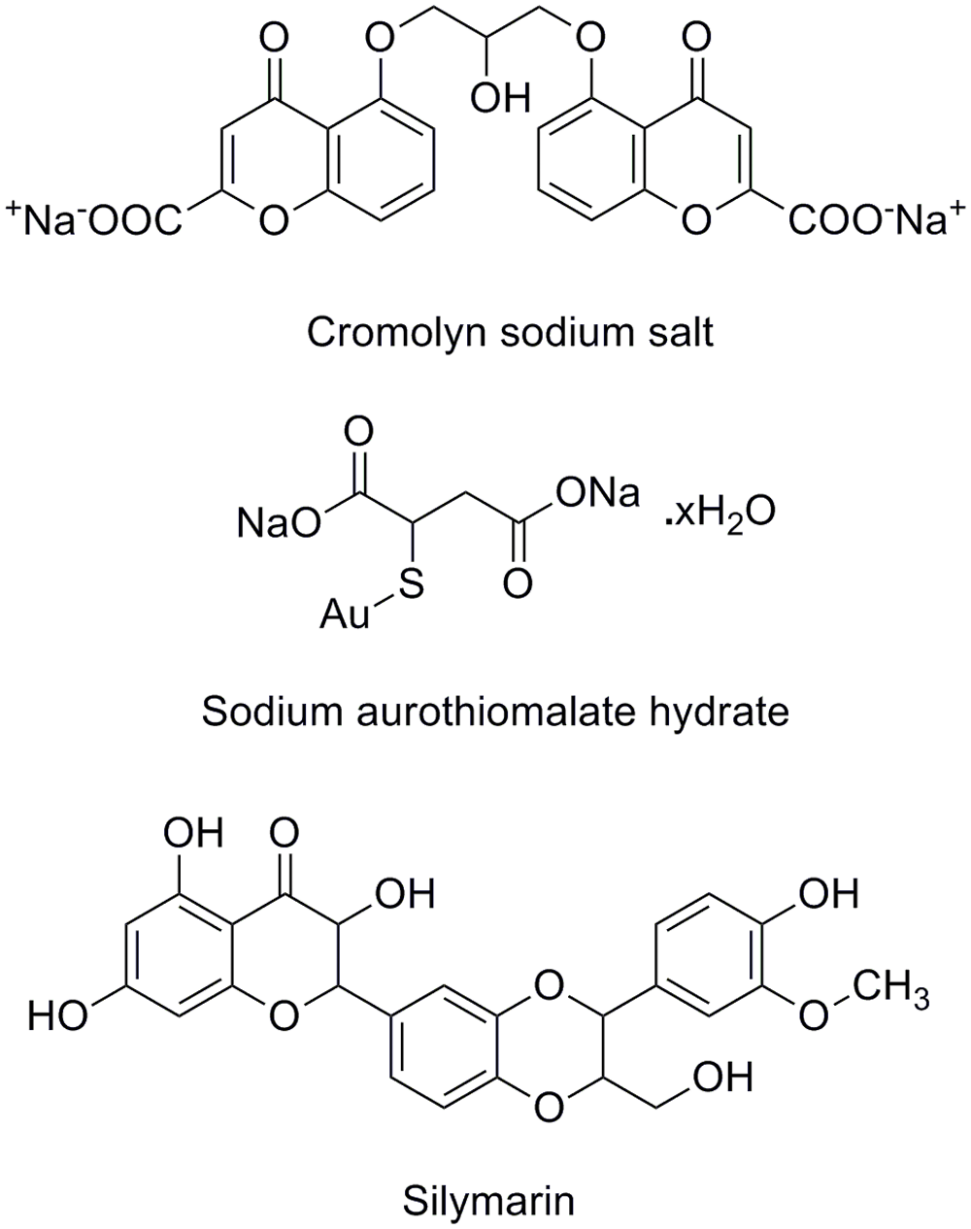
Hyaluronidase inhibitors. Cromolyn sodium salt, sodium aurothiomalate hydrate, and silymarin.

**Fig 5:**
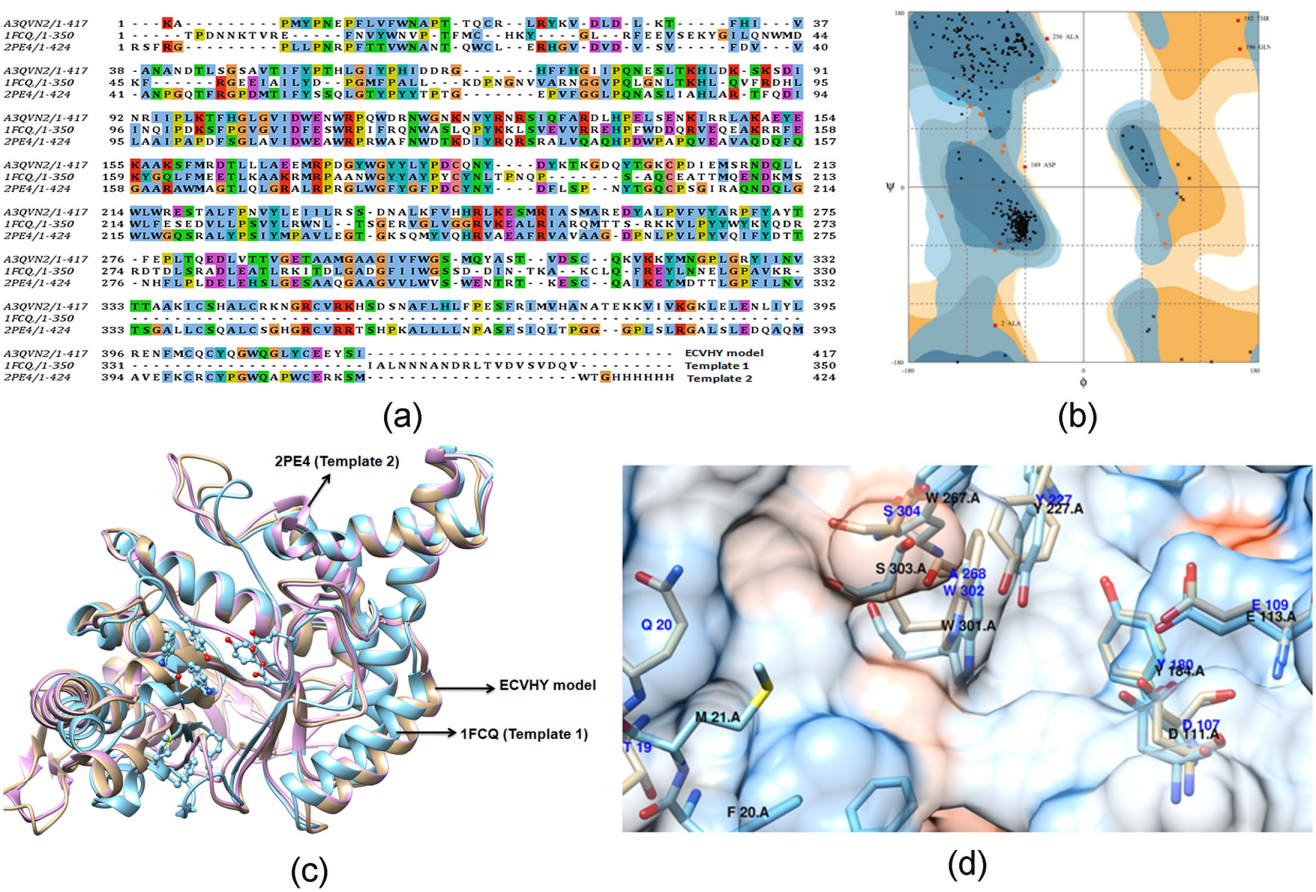
Predicted structure of ECVHY. (a) Target—template sequence alignment, (b) model validation, (c) target-template structure superposition and (d) conserved active site residues. The template structures—bee venom hyaluronidase (PDB ID: 1FCQ-template 1) and human hyaluronidase (PDB ID: 2PE4- template 2) showed 33.3% and 42% sequence identity and 92% and 70% query coverage with the target sequence—*Echis ocellatus* venom hyaluronidase (UniProt ID: A3QVN2).

**Fig 6:**
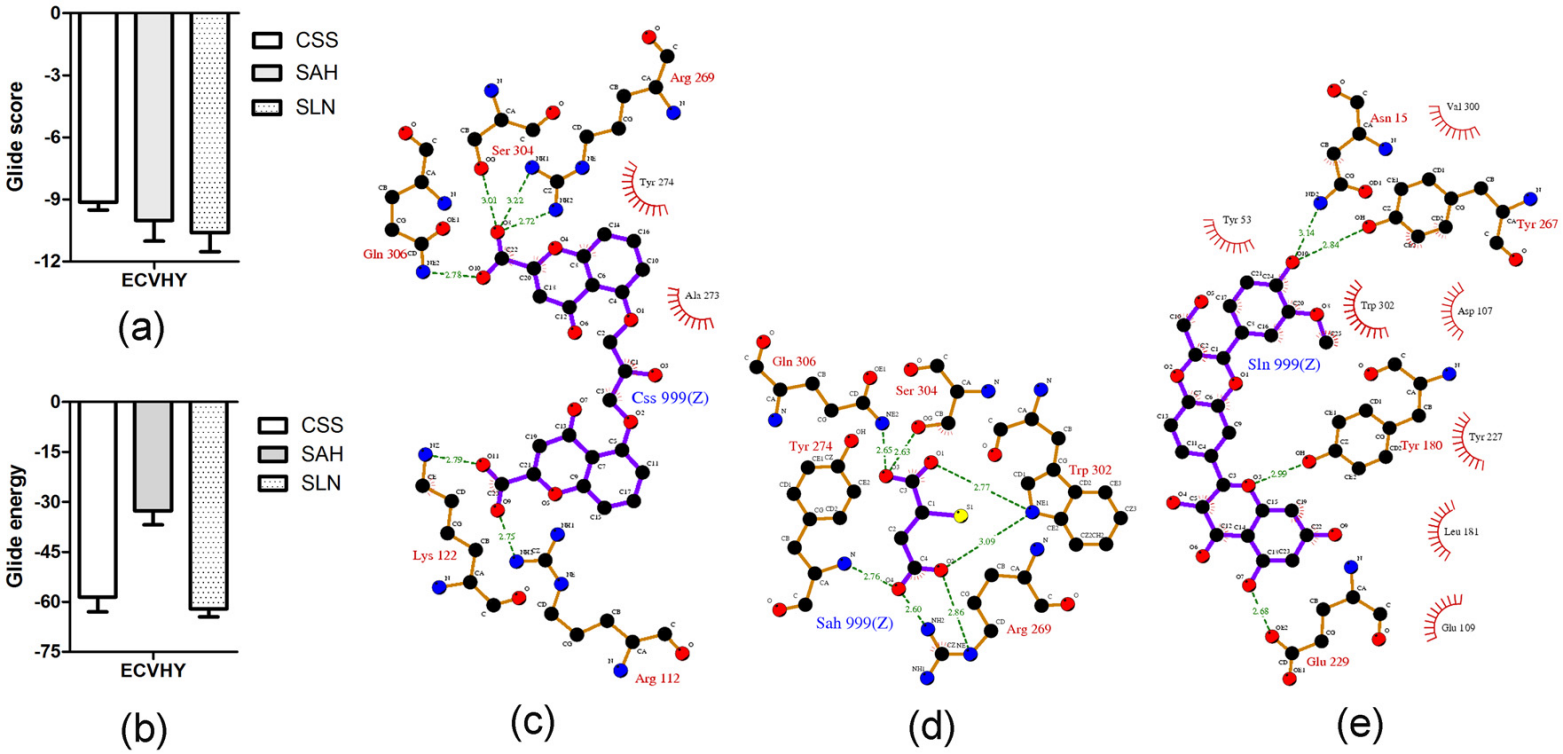
Energetically favorable binding modes of CSS, SAH, and SLN calculated using IFD method. Glide score (a) and glide energy (b) (calculated in kcal/mol) associated with best binding modes of CSS, SAH, and SLN with the active site of modeled ECVHY. The hydrogen bonding and hydrophobic interactions between the enzyme and CSS (c), SAH (d), and SLN (e) respectively are depicted using the LigPlot software. CSS, SAH, and SLN are labeled using respective three letter codes with a common residue number 999(Z).

Among known hyaluronidase inhibitors, CSS and SAH are reported as potent inhibitors against SVHYs induced local tissue necrosis in independent type experiments [39]; SLN is known for its varied pharmacological applications including inhibitory potentials against elevated serum hyaluronidases in arthritic individuals [40]. Based on this information, CSS (PubChem ID: CID 2882), SAH (PubChem ID: CID 6268) and SLN (PubChem ID: CID 31553) (Fig 4) were prepared and screened for their inhibitory potentials against modeled 3D structure of ECVHY (Fig 5). Among the screened inhibitors, SLN showed promising IFD results with lower glide score (-10.60 ± 0.92 kcal/mol), glide energy (-62.04 ± 2.5 kcal/mol) values and stable hydrogen bond (Tyr180, Tyr267, Asn15 and Glu229), hydrophobic (Leu181, Try227, Try53, Trp302, Asp107, Val300, and Glu109) interactions with ECVHY compared to CSS and SAH (Fig 6).

### Comparative evaluation of AP and SLN against ECVPLA_2_s and ECVHYs

**Fig 7:**
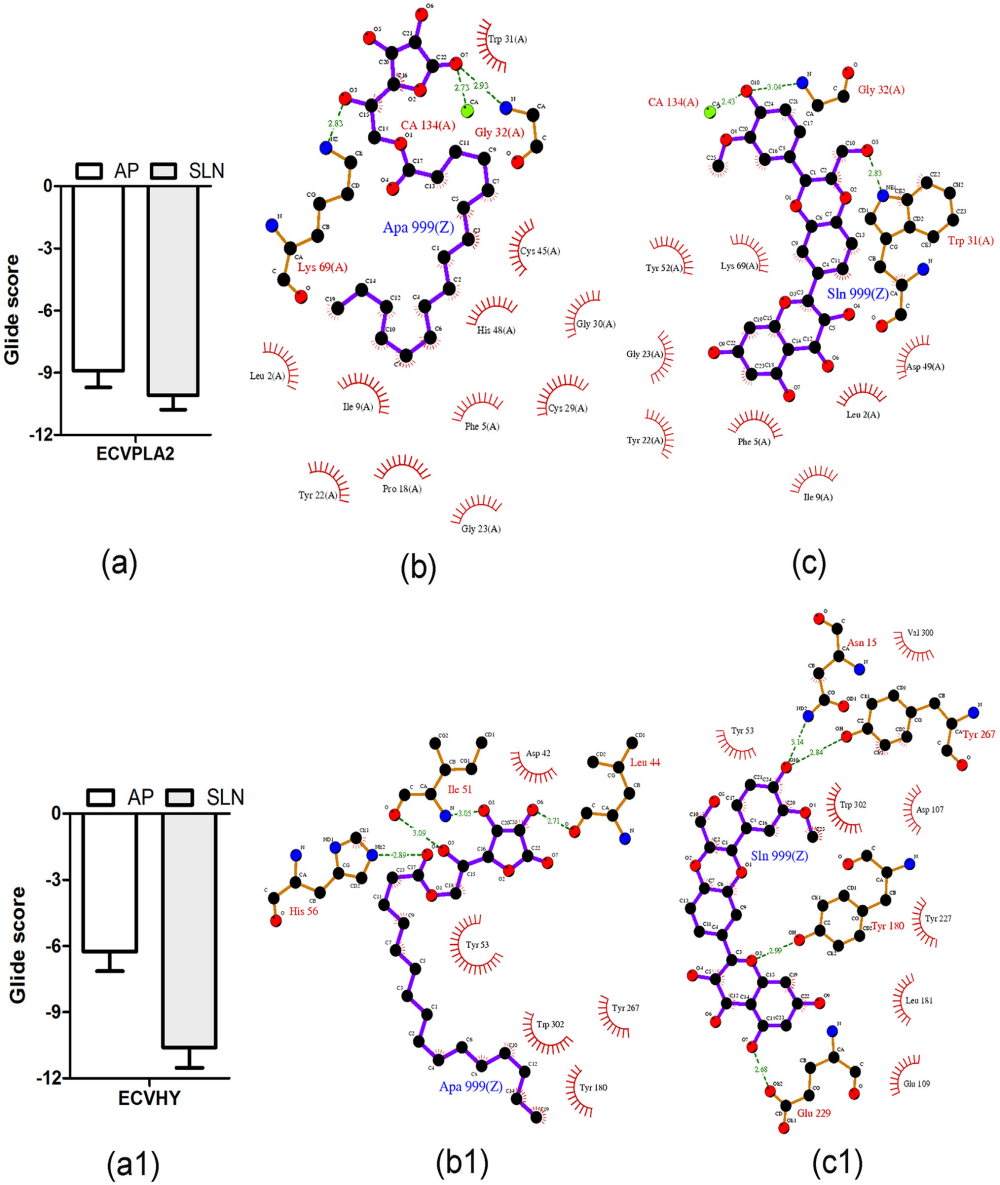
Energetically favorable binding modes of AP and SLN calculated using Induced fit docking method. Glide score (calculated in kcal/mol) associated with best binding modes of AP and SLN with the active site of ECVPLA_2_ (a) and modeled ECVHY (a1). The hydrogen bonding and hydrophobic interaction of AP and SLN with ECVPLA_2_ (b, c) and modeled ECVHY (b1, c1) respectively are depicted using the LigPlot software.

On the basis of computational studies AP and SLN were chosen as potent ECVPLA_2_s and ECVHYs inhibitors respectively. However, the lipophilic derivative of ascorbic acid (AP) is proved to be one of the most potent inhibitors of hyaluronidases, particularly against streptococcal and bovine testicular hyaluronidase [41] and it shows good inhibition against sPLA_2_. Similarly SLN is known for effective antioxidant and free radical scavenger properties [42], while it shows good hyaluronidase inhibition. This prompted us to comparatively evaluate the dual inhibitory potentials of AP and SLN against ECVPLA_2_s and ECVHYs. Up on docking studies both AP and SLN showed different yet stable hydrogen and hydrophobic interactions with active site residues of ECVPLA_2_ and ECVHY. However, the glide score of SLN (-10.08 ± 0.7 kcal/mol; -10.60 ± 0.9 kcal/mol) was 1.2 and 1.7 times better than that of AP (-8.9 ± 0.8 kcal/mol; -6.26 ± 0.9 kcal/mol) against ECVPLA_2_ and ECVHY respectively. The low glide score of SLN compared to AP on priority suggested SLN as a potent inhibitor towards tested enzymes (Fig 7).

**Fig 8:**
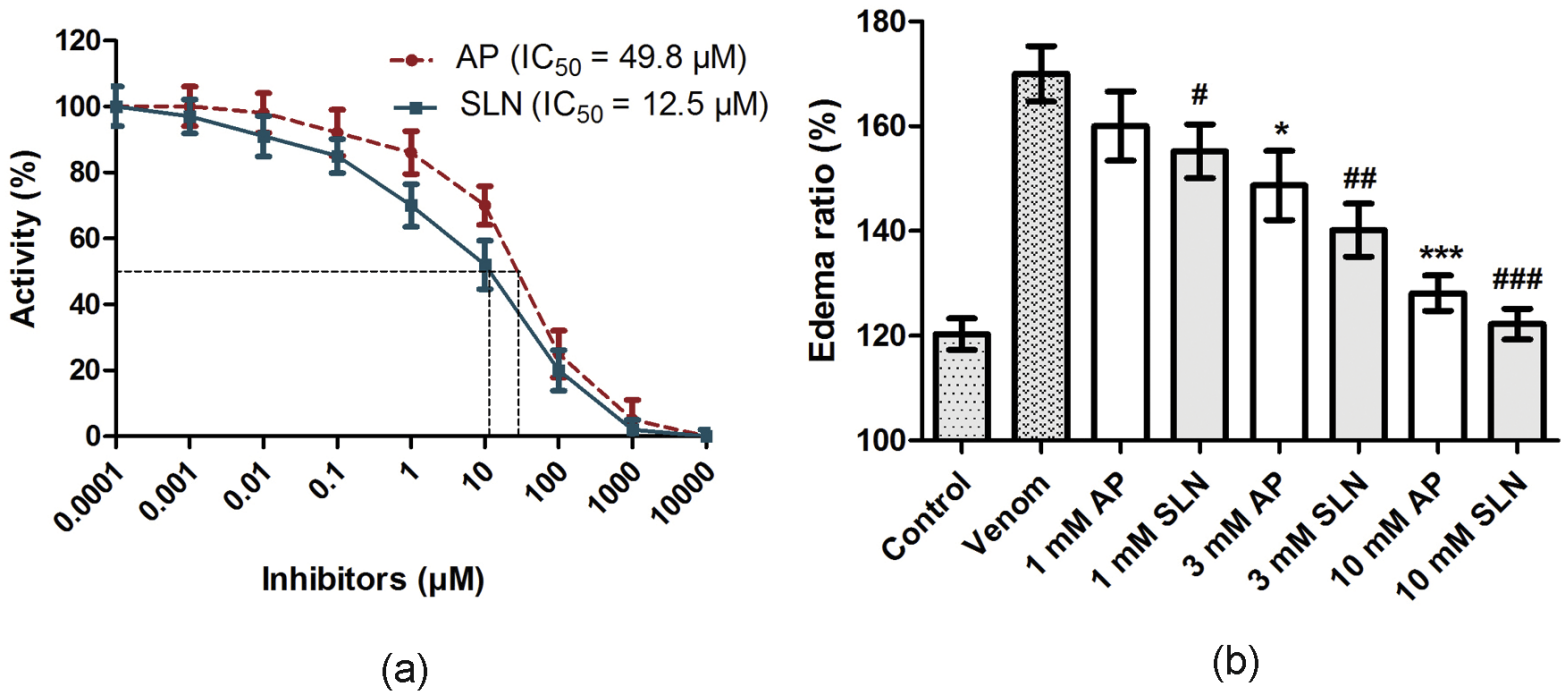
**(a) Inhibition of phospholipase A2 activity of ECV by AP and SLN.** The reaction mixture (350 μl) contained enzyme in 100 mM Tris HCl pH 7.4, 5 mM CaCl_2_ and various concentrations of AP and SLN (0.1 nM-10 mM). The reactions were initiated by adding 30 μl substrate and incubated at 37°C for 45 min. **(b) Inhibition of edema-inducing activity of ECV by AP and SLN:** Mouse intra plantar surface (footpad) was injected with constant 3 μg ECV + various concentrations of AP and SLN following 15 min of ECV injection. After 45 min, the mice were euthanized and both hind limbs were removed at the ankle joint and weighed individually to calculate the edema ratio. *, ^#^ *p* < 0.05, ^##^ *p* < 0.01, and ***, ^###^ *p* < 0.001 compared to ECV induced edema ratio.

The computational outcomes of AP and SLN against ECVPLA_2_ were further evaluated *in vitro* using ^14^C-oleate labeled autoclaved *E*. *coli* as substrate. Both inhibitors significantly inhibited the PLA_2_ activity of ECV in a concentration dependent manner and their IC_50_ values were found to be 49.8 μM and 12.5 μM for AP and SLN respectively (Fig 8a); SLN was ~4 times more potent than AP in inhibiting the activity of ECVPLA_2_s. Cell or plasma membrane is the primary site of action of SVPLA_2_s, where catalytically active forms hydrolyze *sn*-2 acyl bond of glycerophospholipids, in a calcium dependent fashion. This ends up in the generation of free fatty acids and lysophospholipids evoking acute inflammatory reactions associated with pain, swelling (edema) and recruitment of inflammatory cells at the bitten site [10, 12, 15]. In this direction, the specificity of AP and SLN towards PLA_2_ was further evaluated by ECV induced edema inhibition studies. Paw edema was induced in mice by intra-plantar injection of ECV. Edema induction was concentration dependent and the minimum edematic dose (MED) was found to be 1 μg. For inhibition studies, percentage increase of 3 μg ECV injected (170 ± 5%) and saline injected (120 ± 3%) limbs compared to their respective un-injected limbs were used as positive and negative controls as per the mathematical calculations indicated in materials and methods. SLN and AP, upon independent injections following 15 min of ECV administration, dose dependently inhibited the edema-inducing effect of ECV. At 10 mM dose both AP and SLN significantly decreased the edema ratio (128 ± 4%; *p* < 0.0005 and 122 ± 3%; *p* = 0.0001 respectively). However SLN was quite effective compared to AP in preventing ECV induced edema (Fig 8b).

**Fig 9:**
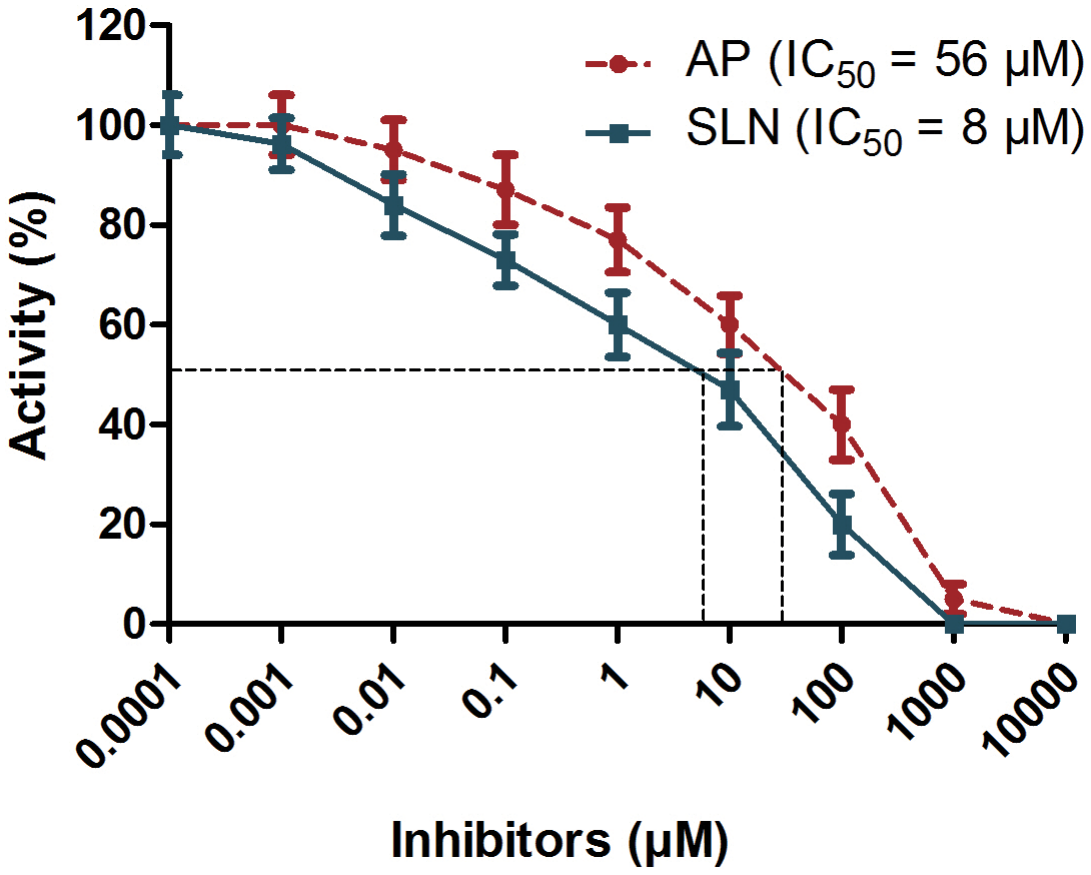
Inhibition of hyaluronidase activity of ECV by AP and SLN. Reaction mixture 300 μl contained 100 μg ECV in 100 mM acetate buffer pH 5.5, 150 mM NaCl and various concentrations of AP and SLN (0.1 nM-10 mM). The reactions were initiated by adding 50 μl substrate (hyaluronic acid) and incubated at 37°C for 2.5 h. After terminating the reaction, the contents were processed for color development.

Snake venom hyaluronidases, though lesser discussed for their role in snake venom pathophysiology, play a key role in producing “spreading response” by hydrolyzing dermal barrier. Hence, the inhibition of these enzymes is crucial to prevent the spread of toxins from the site of bite to systemic circulation [11]. Thus, the inhibitory potentials of AP and SLN towards ECV hyaluronidases activity were estimated by colorimetric assay. Both AP and SLN significantly inhibited the ECVHYs activity in a concentration dependent manner with IC_50_ values of 56 μM, and 8 μM respectively (Fig 9); SLN was 6 times more potent than AP in inhibiting the ECVHYs activity.

### Formulation of inhibitor cocktail of TPEN and SLN and its evaluation against ECV induced hemorrhage and myotoxicity upon independent injection experiments

**Fig 10:**
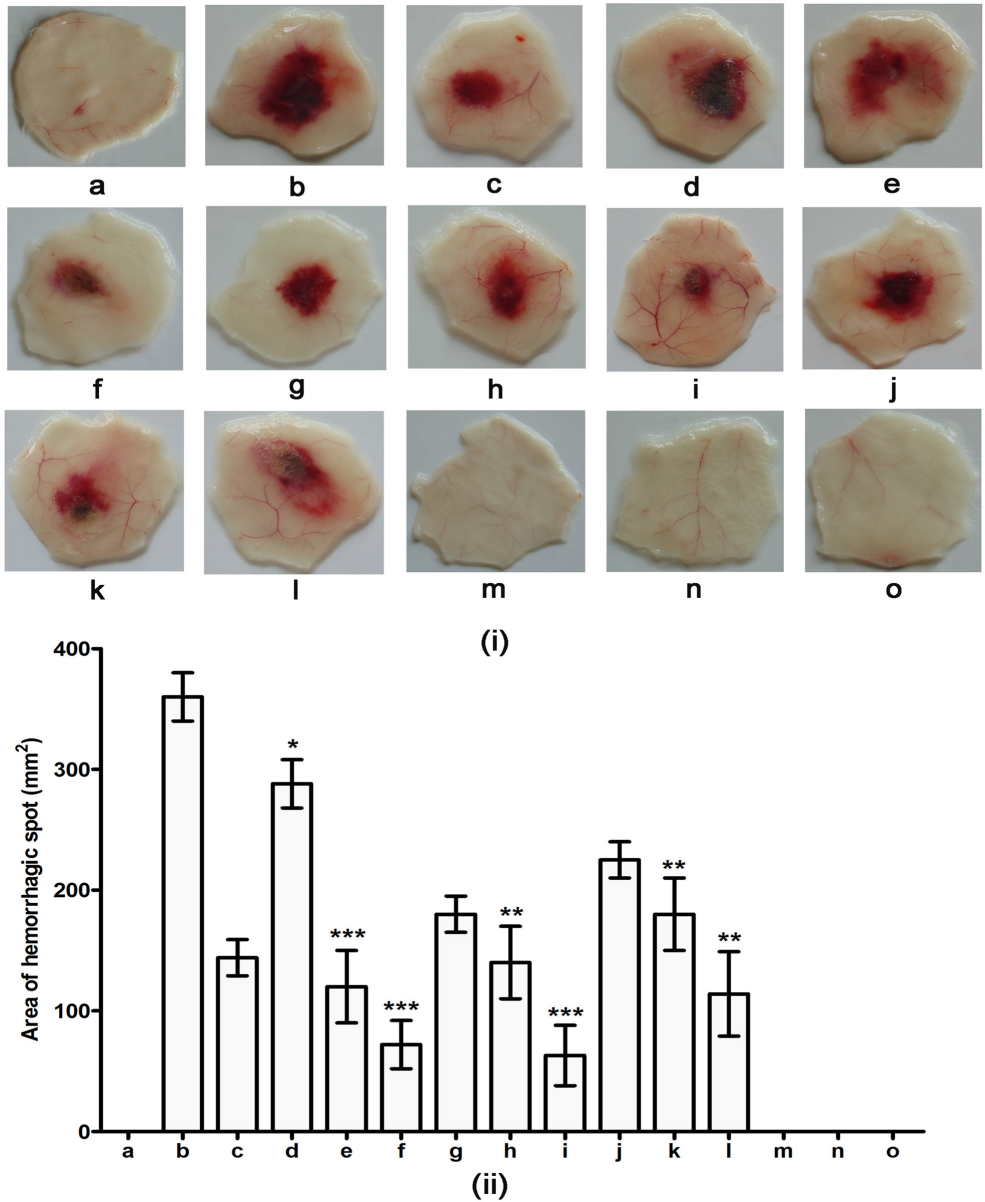
Hemorrhagic activity of ECV and its protection by inhibitor cocktail of TPEN and SLN upon independent injections. (i) Dorsal surface of mouse skin showing hemorrhagic spots. (ii) Area of hemorrhagic spots measured using graph paper. Mice were injected intradermally with constant 3 μg ECV (3 MHD dose) and various doses of inhibitor cocktail of TPEN and SLN (0.3 to 10 mM) at different time points (15 to 60 min) after venom injection. After 3 h, mice were sacrificed and hemorrhagic spots on the inner surface were examined for protection of ECV induced hemorrhage; a. negative control (30μl Saline); b. positive control (hemorrhagic spot appeared after 3 h of 3 μg ECV injection); c. hemorrhagic spot appeared after 15 min of 3 μg ECV injection; d, e, and f: 0.3, 3, and 10 mM inhibitor cocktail injected after 15 min of ECV injection; g. hemorrhagic spot appeared after 30 min of 3 μg ECV injection; h and i: 3 and 10 mM inhibitor cocktail injected after 30 min of ECV injection; j. hemorrhagic spot appeared after 60 min of 3 μg ECV injection; k and l: 3 and 10 mM inhibitor cocktail injected after 60 min of ECV injection; m, n and o: 0.3, 3 and 10 mM inhibitor cocktail alone (cocktail control). *p < 0.05, **p < 0.01, and ***, ^###^ *p* < 0.001 compared to ECV induced hemorrhage.

On the basis of comparative evaluation results of AP and SLN towards ECVPLA_2_s and ECVHYs, on priority SLN was retained for the formulation of inhibitor cocktail along with TPEN. Appropriate molar concentrations of inhibitor cocktail was formulated as described in materials and methods and used for the protection of progressive hemorrhage and myotoxicity. ECV induced hemorrhagic lesion was obtained 3 h after intra dermal injection of crude ECV. The minimum hemorrhagic dose (MHD) was found to be 1 μg. Three MHD was used for inhibition studies as it could progressively increase the area of hemorrhagic spot time dependently. ECV induced the hemorrhagic lesion of 40 ± 4% at 15 min, 50 ± 5% at 30 min, and 63 ± 5% at 60 min respectively compared to hemorrhagic spot formed at the end of 180 min (100%). Initially different molar concentrations (0.3 mM, 3 mM, and 10 mM) of inhibitor cocktail (containing 1:1 molar concentrations of TPEN and SLN respectively) was tested following 15 min of ECV administration. Among the tested doses 3 mM (65 ± 15% inhibition; *p* < 0.0005) and 10 mM (80 ± 22% inhibition; *p* < 0.0001) inhibitor cocktails significantly prevented the progression of hemorrhage. On the basis of results obtained 3 mM and 10 mM inhibitor cocktails were further used for hemorrhage inhibition studies following 30 and 60 min of ECV administration. At tested doses and time points inhibitor cocktail considerably protected the progression of hemorrhage. The results were statistically significant with 61 ± 13%; *p* = 0.0005 inhibition for 3 mM and 82.5 ± 30%; *p* < 0.0001 inhibition for 10 mM at 30 min compared to 50 ± 8%; *p* < 0.001 inhibition for 3 mM and 68 ± 20%; *p* = 0.0005 inhibition for 10 mM at 60 min respectively (Fig 10).

**Fig 11:**
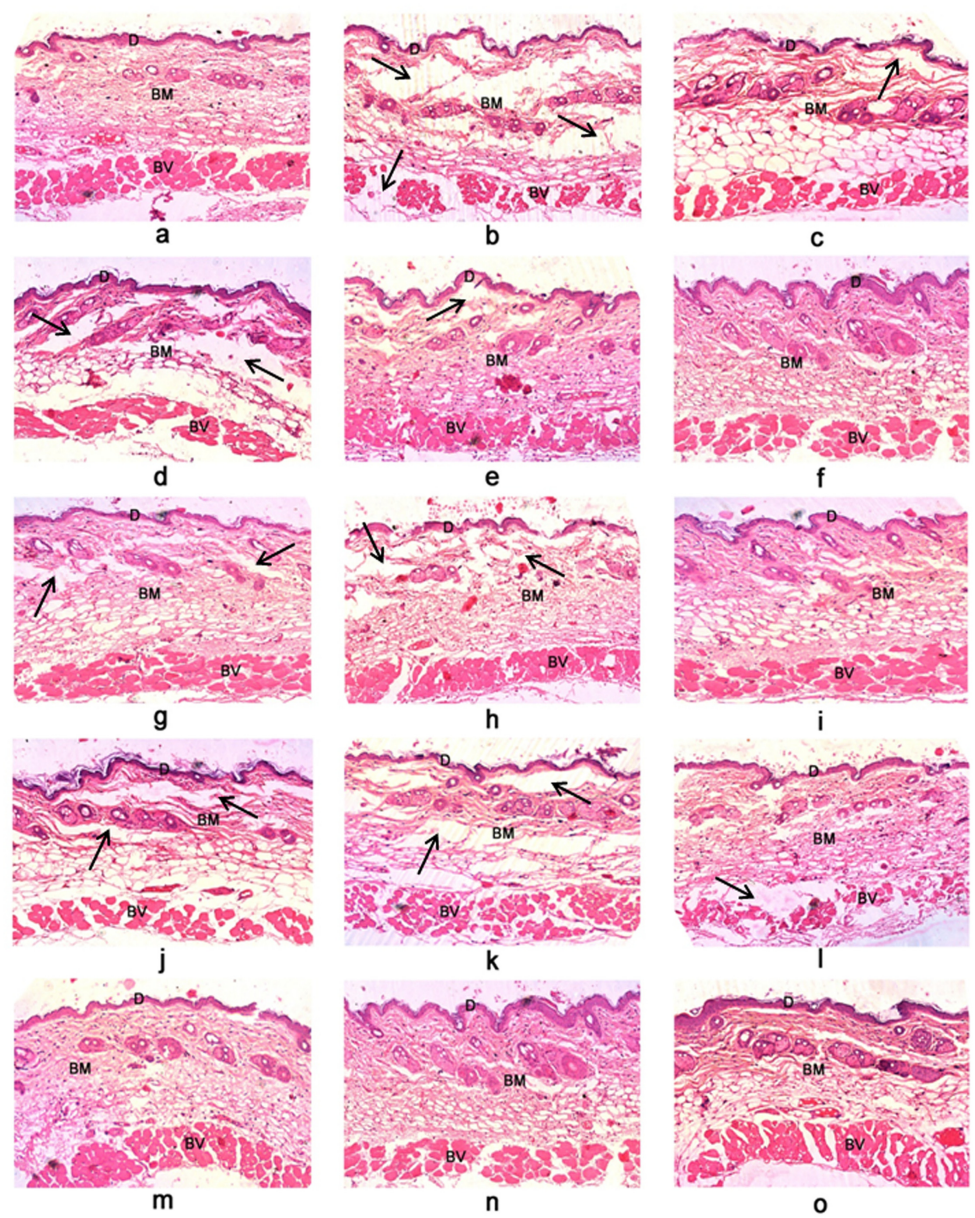
Photomicrographs of mice skin transverse sections observed at 100 X magnification showing protection against ECV induced hemorrhage by inhibitor cocktail. (a) Saline-injected control section showed intact dermal layer (D), basement membrane (BM) and surrounding blood vessels (BV). 3μg ECV injected sections dissected at different time points—15 min (c); 30 min (g); 60 min (j); and 180 min (b) showed disorganized dermis, basement membrane and disruption of blood vessels in time dependent fashion. On independent injection (following 15, 30, and 60 min of ECV administration) inhibitor cocktail showed dose-dependent protection against venom-induced hemorrhage—(d), (e), (f): 0.3, 3, and 10 mM inhibitor cocktail injected after 15 min of ECV injection; (h) and (i): 3 and 10 mM inhibitor cocktail injected after 30 min of ECV injection; (k) and (l): 3 and 10 mM inhibitor cocktail injected after 60 min of ECV injection. Cocktail control—(m), (n) and (o): 0.3, 3, and 10 mM inhibitor cocktail alone injected sections showed intact ECM and the basement membrane surrounding the blood vessels. The dark arrow represents the degraded portions of tissue sections.

The above results were supported by histological examination of skin sections. The skin section of ECV injected hemorrhagic spot showed extensive damage of dermis, basement membrane surrounding blood vessels and massive infiltration of inflammatory cells. Saline and inhibitor cocktail alone injected skin sections showed intact dermis and basement membrane. Further no infiltration of inflammatory cells was observed. Treatment groups showed reduction in tissue damage and infiltration of inflammatory cells (Fig 11). In our previous study we have shown the protection of progressive hemorrhage by 20 mM TPEN upon independent injection following 15 min of ECV administration (90.2 ± 10% inhibition; *p* < 0.0001). However, in this study we documented such hemorrhage protective effect with 3 mM inhibitor cocktail (80 ± 22% inhibition; *p* < 0.0001). Further, we have shown the protection of ECV induced progressive hemorrhage for succeeding time intervals (30 and 60 min following ECV administration). This clearly substantiates the efficacy of mixture of inhibitors which are specific and can work in a combinatorial fashion against progressive hemorrhage induced by mixture of toxins in crude venom.

**Fig 12:**
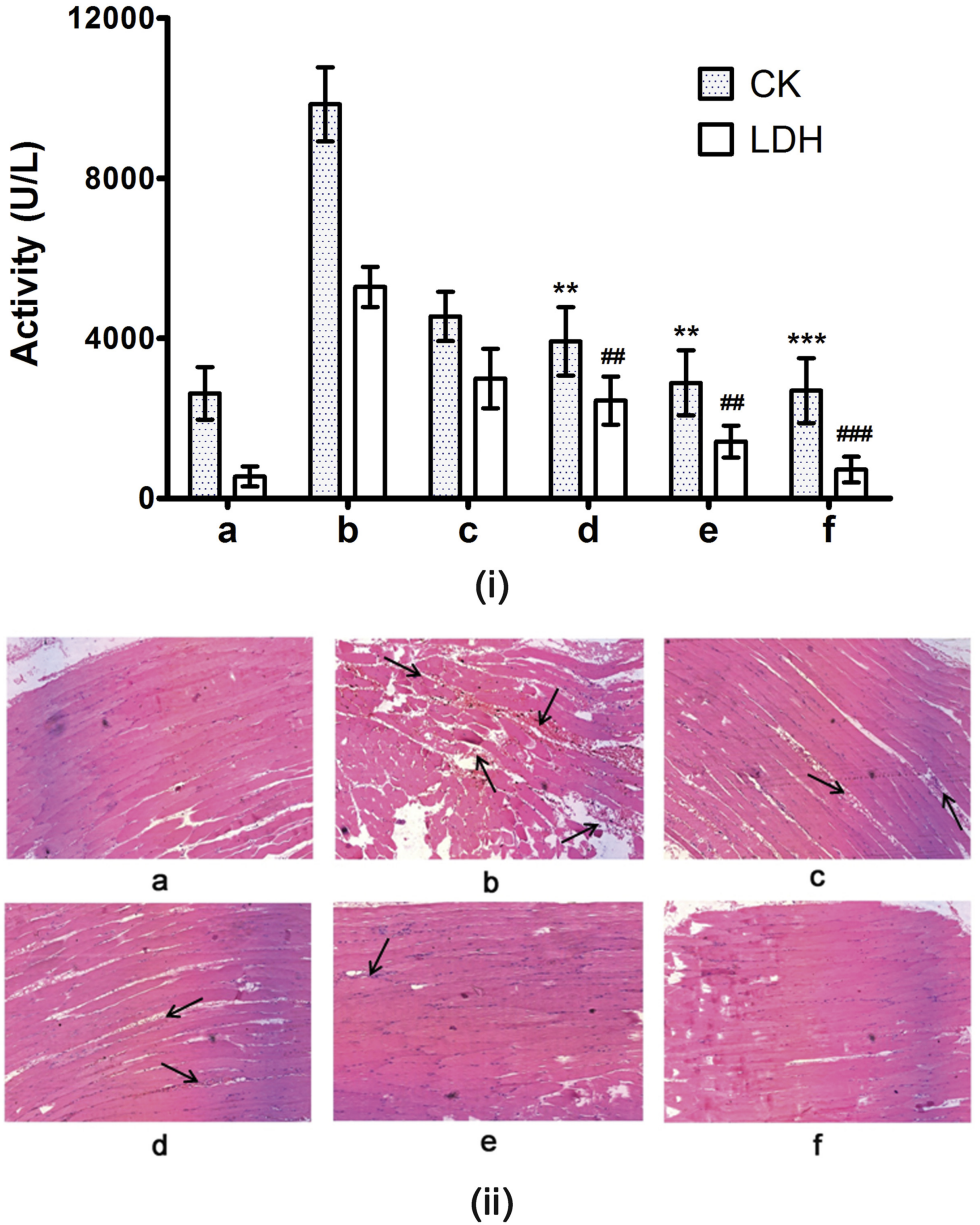
(i) Serum creatine kinase (CK) and lactate dehydrogenase (LDH) levels and (ii) histopathology of mice injected (i.m.) with ECV and its protection by inhibitor cocktail. Mice were injected with 5 μg ECV + different doses of inhibitor cocktail (independently after 30 min of ECV injection). After 3 h, mice were sacrificed and serum CK and LDH levels were assayed using AGAPPE kit. **, ^##^ *p* < 0.01 and ***, ^###^ *p* < 0.001 compared to ECV induced CK and LDH values. Further, dissected thigh muscles from the site of ECV injection were processed for hematoxylin and eosin staining and were observed at 200 X magnification. (a) Saline control showed characteristic muscular striations and intact myocytes. Five μg ECV injected sections dissected at different time points—30 min (c); and 180 min (b) showed disorganization in muscular striations and myocytes in time dependent fashion as evidenced by proportionate elevation of serum CK and LDH activities compared to control. On independent injection, inhibitor cocktail—(d), (e): 3 and 10 mM showed dose-dependent protection against ECV induced myotoxicity. 10 mM inhibitor cocktail alone-injected section (f) showed characteristic muscular striations and intact myocytes. The dark arrows show the damaged portion of muscle sections.

The progressive hemorrhage induced by orchestrated actions of Zn^2+^SVMPs, myotoxic SVPLA_2_s and SVHYs further leads to severe tissue damage—dermo and myo-necrosis; which in turn depends on the concentration of these hydrolytic enzymes in crude venom [14, 43]. SVMPs results in ECM, vascular degeneration and ischemia; myotoxic SVPLA_2_s (enzymatic/non-enzymatic) exert direct action of on the plasma membrane of muscle cells and tissues; SVHYs rapidly degrade hyaluronic acid. Overall, the combinatorial action of these enzymes causes decreased viscosity at the envenomed site and lead to progressive myotoxicity [8, 10, 44]. Following damage to the skeletal muscles, creatine kinase (CK) and lactate dehydrogenase (LDH) are released into serum, resulting in their elevated levels. Thus, ECV induced myotoxicity was quantified by elevated serum levels of CK and LDH (9, 847 ± 924 U/L and 5, 289 ± 500 U/L respectively) against corresponding values in serum of control mice (2, 628 ± 653 U/L and 550 ± 250 U/L respectively). For inhibition studies, 3 and 10 mM doses of inhibitor cocktail were injected 30 min post envenomation in separate groups of mice. Inhibitor cocktail of TPEN and SLN dose dependently protected the increase of serum CK and LDH levels, and significant effect was observed at 10 mM dose (2, 889 ± 813 U/L; *p* = 0.0007 and 1, 425 ± 400 U/L; *p* = 0.0005 for CK and LDH respectively). The variations in CK and LDH values in control, ECV and inhibitor cocktail treated groups were supported by microscopic observations of thigh muscle tissue sections. ECV treated groups showed muscle necrosis and accumulation of inflammatory cells at the site of ECV injections in comparison with intact, elongated and striated appearance of control and 10 mM inhibitor cocktail injected groups (Fig 12). The myotoxicity inhibitory potentials of 10 mM inhibitor cocktail administered following 30 min of ECV injection (71 ± 16%; *p* = 0.005 and 80 ± 20%; *p* < 0.005 for CK and LDH respectively) was comparable to that of 20 mM TPEN administered following 15 min of ECV injection (70 ± 15%; *p* = 0.001 and 84 ± 45%; *p* = 0.001 for CK and LDH respectively). However, the inhibitor cocktail was twice more potent than TPEN in terms of molar concentration and it extended the inhibitor administration time to 30 min supporting the hypothesis of combinatorial approach.

## Discussion

Snakebite is a serious health concern in many regions of the world, particularly in tropical and subtropical countries including India as it accounts for large number of mortality and morbidity among the rural population in these areas [45]. In view of geographical, seasonal and species variation of venom toxins, treatment and management of snakebite with classical polyvalent ASV is a major hurdle and the therapy demands for specific ASV [46]. However, even with specific ASV, neutralization of severe local toxicity induced by viperbite is a challenge. *E*. *carinatus* is one among the “BIG FOUR” venomous snakes of India and accounts for large number of mortality in Indian subcontinent [46]. In addition, it induces severe local toxicity characterized by extensive hemorrhage, hemorrhagic edema, blister formation, localized death of dermal and muscle cells leading to progressive dermo and myo-necrosis that persist even after treatment with ASV [12, 47].

Orchestrated action of locally acting, cell and/or matrix degrading principle toxins (Zn^2+^MPs, PLA_2_s and HYs) present in ECV [7] are mainly responsible for severe local toxicity of *E*. *carinatus* bite. In addition, they are also known to trigger remarkable amounts of reactive oxygen/nitrogen species—the key players of oxidative stress and vital organ damage [48]. Thus, concomitant inhibition of these enzymes/toxins alongside ASV is a rate limiting step in viperbite management. Over last decade, several studies have been reported the use of plant extracts, phytochemicals and clinically approved drugs to neutralize viper venom induced local toxicity via pre-incubation method. However, few demonstrate the neutralization of local/systemic toxicity both by co and independent injection experiments using murine model [7, 15, 49].

Although, mono specific inhibitors (phytochemicals and synthetic drugs) are highly effective against crude venom on pre incubation experiments (*in vitro* and *in vivo*), they often fail to tackle the progressive tissue damage induced by mixture of toxins in crude venom upon independent injection experiments [15]. Knowing the limitations of mono specific inhibitors against local toxicity induced by mixture of toxins in crude venom, an ideal synergistic combination of inhibitors is valuable. Specific combination of inhibitors acts via combinatorial fashion to efficiently neutralize the overall toxic effects of the venom in general and progressive local tissue damage in particular. However, selection of ideal synergistic combination of inhibitors to tackle snake venom induced progressive tissue damage is a tricky aspect, as it requires plenty of experimental animals for screening studies. Thus, to overcome constrain of animal ethics and to cut down the number of experimental animals, computational studies serve as better alternatives. Computational studies facilitate high-throughput screening of large number of compounds to narrow down and select potent ones against the protein/enzyme of interest based on their glide score, glide energy and steady interactions with active site residues [16, 50]. Moreover, computational studies are almost comparable to that of *in vitro* and *in vivo* findings [51]. Thus, selection of potent inhibitor against ECVPLA_2_s and ECVHYs among the screened inhibitors using IFD has been detailed in this study. Further, we have demonstrated the efficacy of inhibitor cocktail containing TPEN and SLN against the progressive tissue damage induced by ECV in independent injection type experiments.

In summary, TPEN—a potent Zn^2+^ECVMPs inhibitor completely neutralized local toxicity of ECV up on pre-incubation. However, its effectiveness gradually decreased with time up on independent injection studies, clearly indicating the specificity of TPEN towards Zn^2+^ECVMPs; and involvement of other cell/matrix degrading enzymes (ECVPLA_2_s and ECVHYs) in the progression and aggravation of tissue damage induced by Zn^2+^ECVMPs. In this direction, potent inhibitors against these hydrolytic enzymes were successfully screened using *in silico*, *in vitro* and *in vivo* methods, and SLN was chosen as common ECVPLA_2_s and ECVHYs inhibitor for the formulation of inhibitor cocktail along with TPEN. As expected, upon independent injection studies following 15 min of ECV administration, 3 mM inhibitor cocktail was equally efficient to that of 20 mM TPEN alone in protecting progressive hemorrhage (combination of TPEN along with SLN increased its efficacy ~7 times). Further, 10 mM cocktail administration following 30 and 60 min of ECV injection efficiently protected progressive hemorrhage. Similarly, myotoxicity inhibitory potentials of 10 mM inhibitor cocktail administered following 30 min of ECV injection was comparable to that of 20 mM TPEN alone administered following 15 min of ECV injection (cocktail increased the efficacy of TPEN ~2 times and doubled the inhibitor administration time). This clearly substantiates the effectiveness of combinatorial approach against progressive tissue damage induced by mixture of toxins in crude venom in treatment mode. Besides, upon evaluating stability, inhibitor cocktail was stable for more than six months and successfully exhibited its inhibitory potentials against ECV induced tissue damage with ~92 ± 2% accuracy compared to freshly prepared inhibitor cocktail. Further, it demonstrated negligible signs of toxicity at tested concentrations as evidenced by histopathology of skin and thigh muscle sections.

Overall, the formulated inhibitor cocktail was highly efficient than TPEN alone in protecting progressive tissue damage induced by ECV with negligible adverse effects. With this it opens up a new avenue of combinatorial drug approach to treat viper venom-induced severe local toxicity alongside ASV. Furthermore, as the experimental results accounts for time elapsed from venom injection to inhibitor cocktail administration, topical application of inhibitor cocktail of TPEN and SLN can be a part of first aid in the management of venomous snake bites, particularly viperid and crotalid bites alongside ASV.
